# Toward the genetic landscape of prostate cancer in India: insights from whole-exome and low-pass whole-genome sequencing of formalin-fixed paraffin-embedded tumor tissues

**DOI:** 10.3389/fsysb.2026.1803649

**Published:** 2026-05-20

**Authors:** Soham Biswas, Bhargavi Rajarathinam, Barkha Khilwani, Gaurav Jalendra, Devendra Sharma, Maneesh Vijavargiya, Rajaguru Aradhya, Abdul S. Ansari, Nidhi Shukla, Gyaneshwer Chaubey, Nirmal Kumar Lohiya, Prashanth Suravajhala, The CA Prostate Consortium of India (CAPCI)

**Affiliations:** 1 Department of Biosciences, Manipal University Jaipur, Jaipur, India; 2 Amrita School of Biotechnology, Amrita Vishwa Vidyapeetham, Clappana, Kerala, India; 3 The CA Prostate Consortium of India (CAPCI), Bioclues.org, Hyderabad, India

**Keywords:** genome sequecing, prostate cancer, somatic mutation, systems genomics, whole exome sequencing

## Abstract

**Introduction:**

Prostate cancer (PCa) is a leading malignancy in men, with global incidence showing geographical variations. In India, PCa burden is increasing, especially in urban areas and for men over 50 years of age. Our collaborative effort with the Cancer Prostate Consortium of India (CAPCI) aims to map the genetic landscape of PCa in India, investigate somatic mosaicism, and assess the utility of low-pass whole-genome sequencing (LP-WGS) in determining PCa pathogenesis.

**Materials and methods:**

We performed variant calling using the CONVEX pipeline on whole-exome sequencing (WES) and LP-WGS samples, categorized by tumor grades.

**Results and discussion:**

ClinVar validation of WES variants identified 12 consensus somatic variants mapped to gnomAD, with 11 being pathogenic, likely pathogenic, or variants of uncertain significance (VUS). We also screened 956 germline variants common to at least two of the 49 samples, identifying 88 common variants, including missense and small InDels. Common and unique somatic variants were found in APC, BRAF, ATM, TP53, SMAD4 and CHEK2 genes. Four of these (rs62619935, rs202160435, rs121913343, and rs141568342) are extremely rare (MAF <= 0.001), seven single nucleotide variants (SNVs) are novel, with four being VUS. Pathogenic somatic variants in higher Gleason score samples (4+4 and 4+3) were homozygous, while benign prostatic hyperplasia (BPH) samples with lower Gleason scores were heterozygous, aligning with population stratification. Whereas the rs1042522 variant in the TP53 gene, with a gnomAD MAF of nearly 38%, was a common somatic SNV, we also identified distinct CAG repeat mosaicism associated with PCa risk.

## 1 Introduction

Prostate cancer (PCa) is among the most commonly diagnosed forms of malignancy affecting men worldwide, and the fifth leading cause of cancer-related deaths. According to the 2022 Global Cancer Observatory (GLOBOCAN) report (Bray et al., 2024), approximately 1.5 million new cases of PCa have been reported worldwide. In the same year, it accounted for around 397,430 deaths, with an age-standardized mortality rate of ∼ 7.3 per 100,000 males. The global incidence exhibits marked geographical disparities, with high rates in North America, Europe, and Australia, while many low- and middle-income regions show lower recorded incidents but a rising trend in mortality (Schafer et al., 2025). In India, cancer surveillance programs, e.g., Population Based Cancer Registries (PBCRs), indicate an increasing PCa burden. In a recent compilation of 43 PBCRs, a staggering 1,562,099 cases of cancer and 874,404 related deaths in India across all possible tumor sites were projected (Mathur et al., 2025). PCa is estimated to contribute ∼ 3% of all new cases in India, with an age-standardized incidence rate (ASR) in urban areas, such as Delhi, Mumbai and Kolkata, falling within a range of 9–12 cases per 100,000 men (Sankarapillai et al., 2024). Notably, in the Indian context, incidence increases steeply after the age of 50 and accelerates beyond the age of 64 (Kumar et al., 2025a; Bhargavi et al., 2023), with a substantial proportion of cases (∼43%) being diagnosed at distant metastatic stage at some registries (Bakshi et al., 2022). While PCa is not among the leading causes of cancer mortality in India, its mortality burden is meaningful in the non-communicable disease landscape (Kumar et al., 2025b). Age is a well-recognized risk factor of PCa incidence and outcomes, and the Indian PBCR analyses support this hypothesis. Descriptive epidemiology from Indian registries reveals that incidence and mortality escalate with advancing age, particularly over 64 years of age (Jain et al., 2014). Whole-exome sequencing (WES) has rapidly become indispensable for identifying PCa-specific somatic and germline alterations, enabling the discovery of prognostic biomarkers and therapeutic targets that are not apparent from clinical features alone. Large cohort-based multi-national WES and transcriptomic studies, including that of CA Prostate Consortium of India (CAPCI)’s efforts on metastatic castration-resistant PCa, demonstrated frequent variations in AR, TP53, PTEN, and DNA-repair genes, viz. BRCA1/2 and ATM (Robinson et al., 2015; Gupta et al., 2020; Breyer et al., 2012).

Deep exome surveys of localized non-indolent tumors indicate that while single-nucleotide driver mutations are less common in metastatic cases, these tumors often harbor recurrent structural and copy number variations (CNVs), such as FOXA1 and MYC, which are associated with disease aggression and recurrence (Taylor et al., 2010; Fraser et al., 2017). Furthermore, whole-genome sequencing (WGS) analyses have identified associations with APC, AR, and ERG genes, along with non-coding variations in microRNAs (miRNAs) and long non-coding RNAs (lncRNAs), as significant contributors to progression and new therapeutic hypotheses (Wedge et al., 2018). Notably, large cohort analyses using both WES and WGS demonstrate that BRCA2-mutant metastatic tumors exhibit distinct genomic loss-of-heterozygosity, a higher tumor-mutational burden, and poorer outcomes on certain therapies compared to ATM-mutant tumors. These findings suggest a strong case for clinical stratification at the gene level rather than focusing solely on pathway-level analysis (Hwang et al., 2023; Pedrani et al., 2025; Frimpong and Teply, 2025).

Over the last 3 years, low-pass shallow whole-genome sequencing (LP-WGS) has been of great use for discerning candidate variants, especially repeat expansions for the desirable phenotypes, which proved to be a cost-effective alternative to WES (Le and Durbin, 2011). The present study, as a part of a collaborative effort of the CAPCI, is aimed at (a) making a genetic landscape for PCa in India, b) wherein we sought to ask whether somatic mosaicism is associated with PCa pathogenesis, and (c) investigating if LP-WGS can provide evidence for determining PCa pathogenesis, and thus we employed LP-WGS as a pilot.

## 2 Materials and methods

### 2.1 Sample cohort

The samples were collected from Rukmani Birla Hospital (RBH) and Mahatma Gandhi University of Medical Sciences and Technology (MGUMST) in Jaipur, India, and all patients were identified as native to North or North-west India. All methods were performed in accordance with relevant guidelines and regulations. Archival pathological Formalin-fixed Paraffin-embedded (FFPE) and Fresh Frozen blocks containing specimens were obtained retrospectively after clearance from the institutional ethics committee of both RBH and MGUMST, and informed consent was duly obtained, wherever applicable. Among FFPE blocks, thirty samples were marked as malignant, and five samples were designated as BPH (Supplementary Table S4). The inclusion criteria for malignancy and BPH were set based on the Gleason grades, with Gleason grade greater than or equal to 6 being malignant, otherwise BPH, while other inclusion and exclusion criteria were accorded previously (Shukla et al., 2023).

### 2.2 DNA extraction, library preparation and sequencing

**FIGURE 1:**
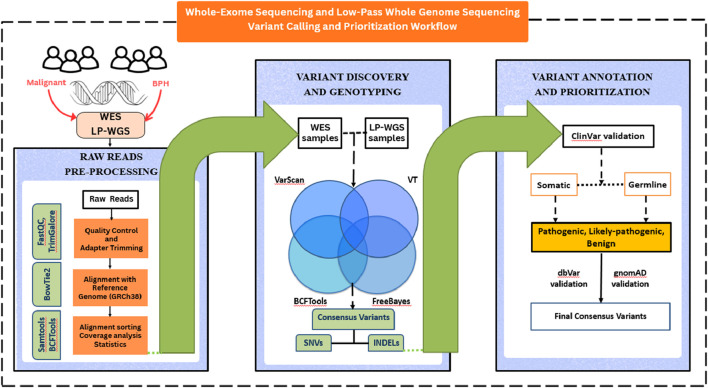
Schematic representation of workflow.

49 malignant adenocarcinoma and BPH FFPE and Fresh-Frozen de-identified samples were collected from the aforementioned hospitals. Samples with a Gleason score between 6–9​ were selected for the study. DNA was extracted using the QIAamp DNA FFPE Tissue Kit; exome library preparation was done using Twist Biosciences 2.0 exome kit (Cat no. 104119). Library preparation and WES were performed at Unipath Speciality Laboratory Ltd., Ahmedabad on a Novaseq 6,000 platform to generate paired-end reads using 150 bp chemistry with at least 110× depth coverage, resulting in 7–8 GB of data per sample (Figure 1). For LP-WGS samples, genomic DNA was extracted from archival FFPE blocks derived from ten samples, wherein five malignant tumors with Gleason scores ≥ 7 and five BPH samples with Gleason scores below 6. The LP-WGS was subjected to these samples, following standard library preparation and quality control, achieving a depth of coverage of 1.6x to 4x. In short, 50 ng of DNA (qubit quantified) was subjected to enzymatic fragmentation, end repair, and addition of poly-A at the 3′-prime end in a single reaction, followed by ligation of universal adapters. After purification using 0.8X magnetic beads (supplied with the kit), unique dual indexes were added to each sample, followed by PCR amplification. All the libraries were purified to remove PCR components used, and subsequently quantified using Qubit 4.0 fluorometer for pooling before the hybridization step. A total of 1,500 ng of pooled library, consisting of 187.5 ng library per sample, was used for an overnight hybridization reaction. In this reaction, millions of biotinylated probes targeting around 20,000 human genes are used to capture exonic regions of the human genome. Post-hybridization, the targeted exonic region (approx.41 mb) is captured using streptavidin beads, followed by subsequent washes to remove unbound probes. Final amplification is performed using Illumina-specific primers, followed by a quality check using HS D1000 screen tape on Agilent tape station 4,150 (Agilent Technologies). The average library size of all the libraries ranged from 330bp to 390bp.

### 2.3 Quality control and data pre-processing

Raw sequencing data were first subjected to quality assessment using FastQC. Metrics evaluated included per-base sequence quality, GC content, sequence duplication levels and adapter contamination. Quality reports for all samples were aggregated and visualized using MultiQC. Specifically for LPS-WGS samples, elevated adapter content was processed with TrimGalore for adapter trimming.

### 2.4 Prioritization of variants

High-quality reads with trimmed adapters were then aligned with the Human reference genome GRCh38 version using the aligner BowTie2 (Langmead and Salzberg, 2012). The aligned reads were retained in SAM format and were sorted based on genomic coordinates and then converted into the binary BAM format, followed by indexing for reduced computational cost. The sorted and indexed BAM files were then processed for the variant calling steps using our previously benchmarked in-house pipeline for whole-exome data, called CONVEX (Raju et al., 2023). The CONVEX pipeline utilizes four variant callers: Varscan, BCFTools, FreeBayes, and Vt. It implements a coordinate-based search strategy to identify consensus variants across callers and samples, extracting only those that match genomic positions across all four tools.

These position-matched variants are further normalized by matching respective alternate alleles before undergoing downstream analysis. By employing these four callers, CONVEX prioritizes the accurate prediction of *bona fide* variants. To capture a true positive variant from the potential artifacts, we used different quality thresholds, e.g., minimum depth DP ≥ 20, genotype quality GQ ≥ 20, and allelic balance (AB) for heterozygotes as ≥ 0.2, for WES. Afterwards, we retained only those variants whose FILTER value was set to “PASS”. Downstream annotation was performed using open-source clinical databases, viz. ClinVar, COSMIC and dbVar with the filtering keywords “Pathogenic”, “Likely-pathogenic”, “Variant of Uncertain Significance”, and “Benign”, for somatic and germline variants separately. The ClinVar variants were then verified against the consensus variants discovered by CONVEX, with the similar position-based search strategy and alternate alleles matching, mentioned above. We perused the recent COSMIC annotations (cosmicweb.org last accessed on 25 March2026) and mapped the variants that are present in malignant samples but not in BPH in addition to somatic and loss of heterozygosity (LoH) variants besides considering ClinVar somatic variants (Tate et al. 2019). Further variants were prioritized based on CADD and PolyPhen scores.

### 2.5 Comparative analyses for estimating SNP effects and statistics

The structural variants were extracted from the original variant call datasets and were analyzed for potential CNVs with publicly available and annotated structural variants in gnomAD and dbVar databases using the ‘*bedtools intersect’* command-line tool (Supplementary Material). On the other hand, the GWAS SNV dataset was downloaded from the Prostate Cancer Association Group to Investigate Associated Alterations in the Genome (PRACTICAL) consortium (https://practical.icr.ac.uk; last accessed on 1 November 2025) Phase III study, which contains 3,841 SNVs exclusively associated with PCa pathogenesis (p-value ≤ 0.05). For downstream analyses with the germline variants that were earlier found as pathogenic, we compared those variants against the PRACTICAL GWAS variants, and only the hits matching with chromosome, location, and reference and alternate alleles were retained, by using an in-house bash script. Further mapping was done between the somatic and germline SNVs and their respective sample-level Gleason score, particularly for the samples where the SNVs are found to be unique. We used an in-house Python script to rank the variants based on their respective occurrence as shared between the samples and unique in each sample. The statistical confidence interval (CI) was calculated for discerning candidate CNVs. Somatic and germline pathogenic variants were found to be associated with malignant and BPH patients with homozygous and heterozygous calls, respectively, before being validated using an integrated Genome viewer (IGV) browser (Thorvaldsdóttir et al. 2013; see Supplementary Material).

## 3 Results

### 3.1 Variant discovery in WES and LP-WGS samples

**FIGURE 2:**
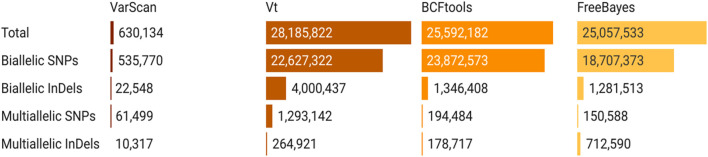
Number of biallelic and multi-allelic variants (SNPs and InDels) called by the four variant callers used in the CONVEX pipeline.

Separate variant-calling steps were performed using the CONVEX pipeline for the WES and LP-WGS samples, segregated by tumor grades. Only the variants matched by positions and alternate alleles from each variant caller were retained and analyzed, with the number of variants called by each of the four variant callers (Figure 2).

The variants called using the CONVEX pipeline on WES samples were subjected to ClinVar validation, wherein PCa-specific annotated variants retrieved from the ClinVar searches yielded 12 consensus somatic variants mapped to gnomAD and Genome India Project (GIP; Bhattacharya et al., 2025) (Table 1). Among them, 11 mutations are pathogenic, likely pathogenic and conflicting, or variants of uncertain significance (VUS), with the *TP53* variant being benign. On the other hand, as many as 956 germline variants were screened common to at least 2 to all 49 samples (Supplementary Table S1).

**TABLE 1:** 12 consensus somatic variants mapped to gnomAD and Genome India Project (GIP).

Chromosome	Position	rsID	Gene	Ref	Alt	ClinVar classification	MAF (gnomAD)	AF (GenomeIndia)
5	112,792,446	rs62619935	APC	C	T	Pathogenic	6.23E-07	NR
5	112,815,507	rs786201856	APC	C	A	VUS	NR	NR
5	112,838,397	rs1170472401	APC	T	C	Likely-benign	NR	NR
7	140,753,334	rs121913364	BRAF	T	G	Likely pathogenic	NR	NR
11	108,247,072	rs202160435	ATM	G	A	Conflicting pathogenicity	7.43E-05	0.000101307
17	7,673,803	rs121913343	TP53	G	A	Pathogenic	3.71E-06	NR
17	7,675,233	rs1597371694	TP53	A	T	VUS	NR	NR
17	7,675,989	rs1555526466	TP53	C	A	VUS	NR	NR
17	7,676,154	rs1042522	TP53	G	C	Benign	0.38	0.521759
17	7,676,230	rs1800371	TP53	G	C/A	VUS	NR	NR/0.000204666
18	51,065,435	rs2144446385	SMAD4	G	T	Pathogenic	NR	NR
22	28,734,532	rs141568342	CHK2	C	T	Pathogenic	1.75E-04	0.000460499

On the other hand, we found common germline variants, *viz.,* rs587779344 (*PMS2)* in as many as 32 samples, whereas rs2518131949 (*CHEK2*) is seen in as few as 2 samples. Taken together, they count to 88 common variants that include missense mutations to small InDels. Common and unique somatic variants were identified in the *APC, BRAF, ATM, TP53, SMAD4* and *CHEK2* genes, out of which four (rs62619935, rs202160435, rs121913343, and rs141568342) variants were found to be extremely rare (i.e., MAF ≤ 0.001), according to gnomAD MAF distribution. These variants are common to genome-wide association studies (GWAS) that were discussed in the methods. In addition, seven single nucleotide variants (SNVs) were not reported in gnomAD, while four were VUS. The pathogenic somatic variants, found in the samples with higher Gleason scores (4 + 4 and 4 + 3), are homozygous, whereas the BPH samples with lower Gleason scores were found to be heterozygous, which is in agreement with the population stratification. The variant rs1042522 (*TP53)* was found to be the common somatic SNV ((NC_000017.11:g.7676154G>C) associated with PCa risk, with a gnomAD MAF ∼ 38% and GIP-AAF > 52%.

**FIGURE 3:**
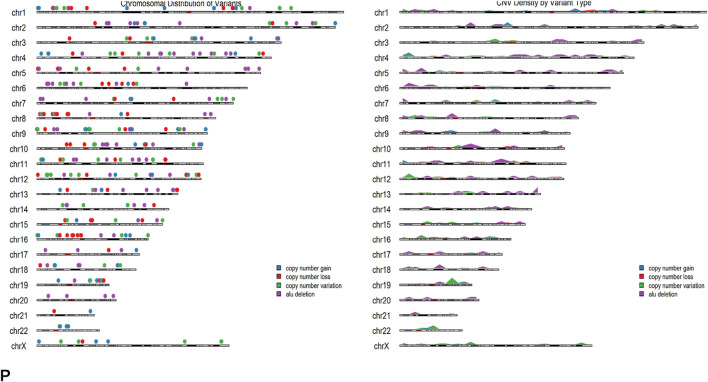
(left panel) Ideogram of *bona fide* CNVs and (right panel) karyogram (see description).

We also observed 3,477 CNVs from as many as 8,406 SVs mapped through the gnomAD *BEDtools intersect* with the NCBI ClinVar, wherein 1778 CNVs were construed to be *bona fide* (Quinlan and Hall, 2010; CI:90% MAF<0.02; Supplementary Table S2; Figure 3). What we sought to ask was whether or not any SNVs could exist within CNVs, which is important for understanding the complete genetic landscape of PCa. Analyzing both could bring complex genetic events that may uncover a previously hidden SNV associated with the disease condition, which might show a combined effect for pathogenesis. However, we could not find any such combined effects, though, as we deem that the complexity of PCa risk could be caused due to the combination of multiple SNVs and CNVs affecting different genes.

### 3.2 Consensus variants from LP-WGS

**FIGURE 4:**
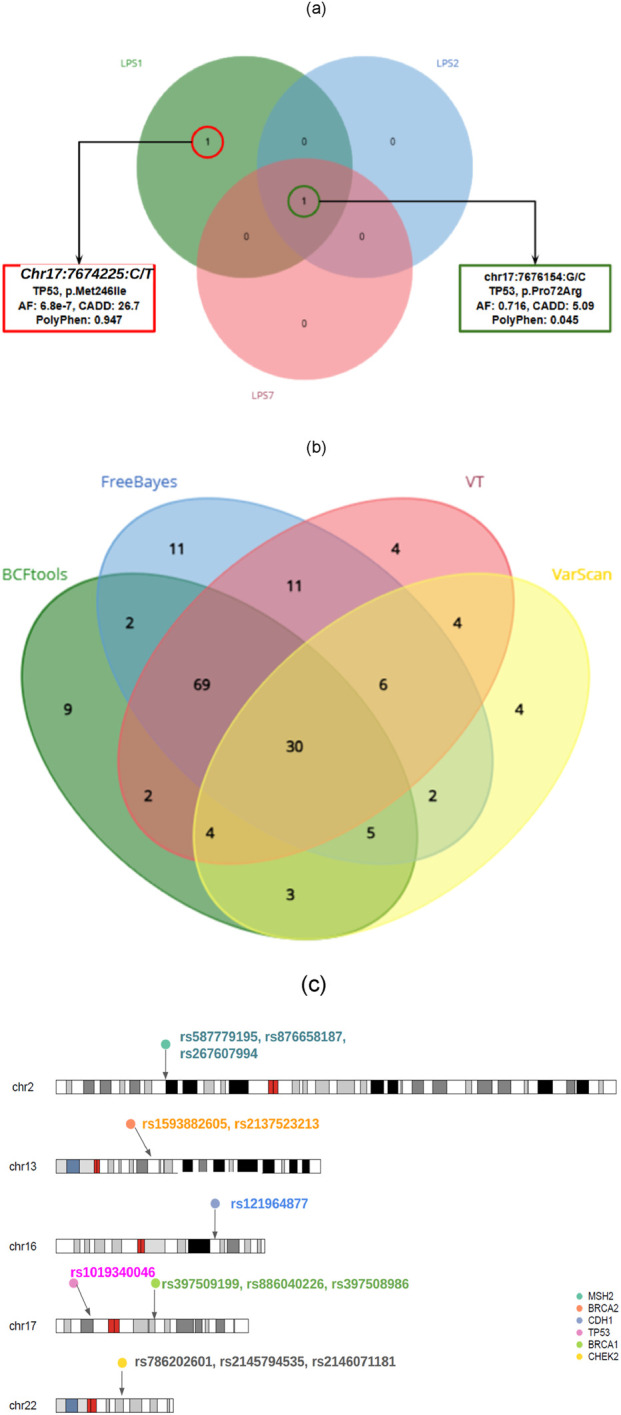
**(a)** Number of consensus somatic variants from LP-WGS, **(b)** Number of consensus germline variants for LP-WGS, and **(c)** Ideogram of 13 pathogenic germline variants found in the LP-WGS samples.

We used the CONVEX pipeline, deploying four conventional variant callers, viz, Varscan, BCFTools, FreeBayes, and Vt, to call both germline and somatic variants in ten LP-WGS (LPS) samples. We found two somatic variants, called by all three variant callers, mapped to ClinVar, and between these two somatic variants, rs1019340046 was found to be pathogenic, present only in the sample LPS1 (Figure 4a). In contrast, the variant rs1042522 was benign in the LPS1, LPS2, and LPS7 samples. When we compared LPS samples individually against both the ClinVar somatic database and previously analyzed WES samples, rs1019340046 associated with *TP53 (NC_000017.11:g.7674225C>T)* was found to be homozygous. On the other hand, we report 30 common germline variants, in which 13 were pathogenic, with 8 common to WES (Figure 4b; Figure 4c). While we reached a consensus, following a search for the potential somatic variant locus in both ClinVar and COSMIC databases for PCa-specific risks or pathogenicity, we could classify variants as somatic (Supplementary Table S5).

### 3.3 Distinct CAG repeats associated with somatic mosaicism

Our analysis showed that shorter CAG repeats (≤18 repeats) are associated with a higher risk of malignant PCa compared to indolent tumors or lower Gleason grades. This suggests that shorter repeat sequences predict higher disease grade. However, we found no association between repeat length variability and low-grade or low-stage disease (Supplementary Table S3), indicating that the clinical implications of a shorter CAG repeat sequence in the androgen receptor (AR) gene warrant further evaluation. We propose an imperative need for genetic tests on PCa biopsy samples, such as those from Radical Prostatectomy (RP), to predict disease progression based on inherent CAG repeats. We also suggest that leveraging pathomics data for these specific samples holds significant unexplored potential. Crucially, integrating these multiomics approaches would allow us to develop machine learning (ML) heuristics for high-dimensional data, providing comprehensive insights into PCa risk through genetic signatures.

## 4 Discussion

PCa is an adenocarcinoma that develops primarily from the glandular part of the prostate gland and commonly metastasizes to the bones and lymph nodes. The aetiology and biological mechanisms for the development of PCa are fairly complex, involving both epigenetic factors and a significant genetic contribution owing to the high heritability of PCa (58%) (Lichtenstein et al., 2000). The major risk factors for PCa are advancing age, family cancer history, germline pathogenic variants, and SNVs associated with an increased PCa risk, ancestry, and mutations in certain genes (Park et al., 2015; Pritchard et al., 2016). The WES serves as a diagnostic odyssey for identifying distinct SNVs associated with PCa risk stratification. Our work exploited checking and identifying common variants associated with PCa risk in India from the latest cohort to that of the pilot study we performed earlier (Gupta et al., 2020). As the work extrapolates to distinct variants, including the somatic mosaicism and heterogeneity associated with tumor burden, we observed that there are specifically CAG repeats that are unique to malignant samples compared to the BPH. What we conceived in our study was that we found the androgen receptor (AR) gene containing a polymorphic CAG repeat in as many as 5 samples (Supplementary Table S3). While shorter CAG repeats have been suggested as a PCa risk factor, recent epidemiologic studies have shown this association, corroborating our results. The somatic AR CAG length may be variable, as we particularly noted the presence of somatic mosaicism in ∼ 36% of cases, which is in agreement with the somatic mosaicism demonstrated by finding different repeat lengths as seen in PCa (Alvarado et al., 2005; Tayeb et al., 2004). We argue that this finding is important as somatic mosaicism is increasingly reported in genetic diseases and is considered a primary source of variable expressivity.

On the other hand, LP-WGS could serve as a cost-effective genome-wide approach for detecting somatic CNVs to assess circulating tumor-DNA (ctDNA) burden. This approach is necessary even though LP-WGS is highly effective for profiling large-scale structural variants (SVs), estimating tumor fractions (TFx), and recovering expected SNVs from plasma or low-input FFPE DNA samples (Hovelson et al., 2017). Conversely, studies in localized PCa have emphasized the low abundance of ctDNA in early tumors and the consequent limits of allele-level SNV detection by LP-WGS alone, arguing for integration with deeper targeted panels or multi-sample statistical calling to recover point mutations (Hennigan et al., 2019). Recent assay-validation and methodological reports confirm that a properly controlled LP-WGS pipeline can robustly call both SNVs and CNVs, supporting targeted therapies for patients and continuous monitoring of progression or response in PCa (Rickles-Young et al., 2024).

We also sought to check the permutations and combinations of discerning variants in the Genome India Project (GIP). The GIP has a published list of alternative allele frequencies (AAFs), not necessarily MAFs. Taken together, we identified variants that are present in malignant samples of CAPCI but absent from large, healthy population databases, such as GnomAD, suggesting that the matched variants are indeed rare and specific to PCa risk, further construing them as strong candidates for pathogenicity. On the other hand, the variants absent from the GIP could be considered strong candidates for being somatic mutations, in addition to rare germline pathogenic variants (Table 1). Nevertheless, absence from our list but mapped to gnomAD/GIP is not sufficient proof of pathogenesis-causing status, as many such variants are benign, particularly in underrepresented populations.

Our work is not free of limitations.

1. We prioritized variants using the Agilent SureSelect Human All Exon V8 kit, as opposed to the V4 version previously used for piloting. The V8 kit offers improved content, uniformity, efficiency, and performance, due to its focused coverage of high-value exonic regions, and regardless of the exome kit version, our variant prioritization utilized a systematic, multi-level filtration and interpretation framework. The only exceptions involved germline variants calling, where predicted effects and inheritance patterns could not be matched due to the lack of familial genotypes.
2. Given the extensive nature of the analyses, we did not use tools such as Exomiser or Alissa to rank variants based on gene-phenotype associations, opting instead for robust and consistent downstream analysis methods. Second, we have noted a significant number of VUS for germline variants mapped to ClinVar (∼18,154). We did not include these VUS in the downstream analyses; however, the pathogenic and likely pathogenic variants that were mapped show a significant association with PCa risk.
3. We did not evaluate variant causality using polygenic risk score (PRS) and Mendelian Randomization (MR) analyses to distinguish *bona fide* variants associated with confounding factors. PRS estimation requires large GWAS discovery cohorts to measure stable SNP effect sizes and on independent validation cohorts to avoid overfitting. Meanwhile, MR requires multiple independent genome-wide significant germline SNPs that satisfy the instrumental variable assumptions, e.g., horizontal pleiotropy, confounding effects, etc. Since our work focused on identifying somatic mutations and a small set of pathogenic germline variants in relatively few genes, it precludes robust PRS construction and the generation of reliable MR predictions.

## Data Availability

The raw reads are available at the Sequence Read Archive (SRA) with the CAPCI project ID: PRJNA616165. The IGV files are available at Supplementary Material: https://drive.google.com/drive/u/0/folders/1ohX1vjS5Yw6FtrdudWJCqi75Xnoae80I. Scripts used for the analyses can be found: https://github.com/Sohambiswas8/PCa_Genetic_Landscape.
