# Supplementary material for: Toward the genetic landscape of prostate cancer in India: insights from whole-exome and low-pass whole-genome sequencing of formalin-fixed paraffin-embedded tumor tissues
Source: Front Syst Biol. 2026 May 20;6:1803649. doi: 10.3389/fsysb.2026.1803649 (PMC13229647; doi:10.3389/fsysb.2026.1803649)
Supplement: Supplementary file 2 [file Supplementaryfile2.pdf]

```
#!/usr/bin/env python3
"""
Collect consensus germline pathogenic variants across samples.

- Input files: all files in the current working directory matching:
    *.vt_germline_pathogenic.txt
  (sample name is derived from the filename prefix before ".vt_germline_pathogenic.txt")

- Key for matching: CHROM:POS:ID
  (ID is taken from a detected ID column in each file; if not found it's set to ".")

- Output: one TSV file `variants_ranked_by_sample_chrposid.tsv` with columns:
    Chromosome, Location, ID, Count, Samples
  Sorted by Count descending (most common -> unique), then chromosome and position.
"""

import pandas as pd
from collections import defaultdict
import os
import re
from pathlib import Path
import sys

# -------- configuration --------
INPUT_GLOB = "*.vt_germline_pathogenic.txt"
OUTFILE = "variants_ranked_by_sample_chrposid.tsv"

# candidate ID column names (case-insensitive)
ID_CANDIDATES = [
    "id", "rsid", "dbsnp id", "dbsnp_id", "dbsnp", "dbsnp_rs", "dbsnp.rs",
    "variant_id", "variant id", "variant", "snp_id", "snpid", "rs_id", "rs-id"
]

# required canonical columns for this dataset
REQUIRED_CHROM = "GRCh38Chromosome"
REQUIRED_POS = "GRCh38Location"

# helper to try reading a file robustly:
#  - try C-engine with tab separator first (fast, default)
#  - if that fails or results in single column, retry with regex whitespace sep using python engine
def robust_read_table(path):
    path = str(path)
    # first try: tab-separated with C engine
    try:
        df = pd.read_csv(path, sep="\t", dtype=str, engine="c")
        # if it reads as a single column, likely wrong delimiter -> retry with regex whitespace
        if df.shape[1] == 1:
            df = pd.read_csv(path, sep=r"\s+", dtype=str, engine="python")
    except Exception:
        # fallback: whitespace-separated using python engine (no low_memory)
        df = pd.read_csv(path, sep=r"\s+", dtype=str, engine="python")
    # normalize column names
```

```
    df.columns = [c.strip() for c in df.columns]
    return df.fillna("")

# -------- helper functions --------

def norm_chrom(c):
    """Normalize chromosome string by removing 'chr' prefix and stripping whitespace."""
    if pd.isna(c):
        return ""
    cstr = str(c).strip()
    return re.sub(r'^[cC][hH][rR]', '', cstr)

def find_id_column(df):
    """Try to find a sensible ID column in dataframe df.
       Returns column name or None."""
    cols = list(df.columns)
    lower_map = {c.lower(): c for c in cols}
    # direct candidates match
    for cand in ID_CANDIDATES:
        if cand.lower() in lower_map:
            return lower_map[cand.lower()]
    # common tokens in header name
    for c in cols:
        lc = c.lower().replace(" ", "_")
        if lc in ("dbsnp", "dbsnp_id", "rsid", "id", "variant_id", "variant"):
            return c
    # name contains 'rs' or 'id' and likely candidate (avoid very long names)
    for c in cols:
        lc = c.lower()
        if ("rs" in lc and len(lc) <= 20) or lc.endswith("id") or lc == "id":
            return c
    # look for values that look like rsIDs in first 50 rows
    for c in cols:
        sample_vals = df[c].astype(str).head(50).tolist()
        for v in sample_vals:
            if re.match(r'^(rs|RS)\d+$', v):
                return c
    return None

def chrom_sort_key(x):
    """Return sort key for chromosomes (numeric chromosomes first in natural order, then X, Y,
others)."""
    try:
        return (0, int(x))
    except Exception:
        xu = str(x).upper()
        if xu == "X":
            return (1, 0)
        if xu == "Y":
            return (2, 0)
        return (3, xu)
```

```
# -------- main --------

def main():
    cwd = Path.cwd()
    paths = sorted(cwd.glob(INPUT_GLOB))
    if not paths:
        print(f"No files found matching pattern {INPUT_GLOB} in {cwd}", file=sys.stderr)
        sys.exit(1)

    print(f"Found {len(paths)} input files. Processing...")

    key_to_samples = defaultdict(set)
    key_to_example_row = {}

    for p in paths:
        fname = p.name
        # extract sample name from filename pattern "<sample>.vt_germline_pathogenic.txt"
        m = re.match(r'^(.*)\.vt_germline_pathogenic\.txt$', fname)
        if m:
            sample = m.group(1)
        else:
            sample = p.stem

        print(f"Reading {fname} as sample '{sample}'")
        try:
            df = robust_read_table(p)
        except Exception as e:
            print(f"Error reading {p}: {e}", file=sys.stderr)
            continue

        # determine chromosome and position column names
        cols_lc = {c.lower(): c for c in df.columns}
        if REQUIRED_CHROM in df.columns and REQUIRED_POS in df.columns:
            chrom_col = REQUIRED_CHROM
            pos_col = REQUIRED_POS
        elif REQUIRED_CHROM.lower() in cols_lc and REQUIRED_POS.lower() in cols_lc:
            chrom_col = cols_lc[REQUIRED_CHROM.lower()]
            pos_col = cols_lc[REQUIRED_POS.lower()]
        else:
            # try a few common alternatives
            alt_chroms = ["chromosome", "chr", "chrom", "grch38chromosome", "grch37chromosome"]
            alt_pos = ["position", "pos", "location", "start", "bp", "grch38location"]
            found_chrom = None
            found_pos = None
            for c in df.columns:
                lc = c.lower().replace(" ", "")
                if found_chrom is None and lc in [a.replace(" ", "") for a in alt_chroms]:
                    found_chrom = c
                if found_pos is None and lc in [a.replace(" ", "") for a in alt_pos]:
                    found_pos = c
            if found_chrom and found_pos:
                chrom_col = found_chrom
```

```
            pos_col = found_pos
        else:
            print(f"File {fname} does not have recognizable chromosome/position columns.",
file=sys.stderr)
            print(f"Found columns: {', '.join(df.columns[:20])}", file=sys.stderr)
            continue

        # find ID column
        id_col = find_id_column(df)
        if id_col:
            print(f"  Using ID column: '{id_col}'")
        else:
            print(f"  No ID-like column found in {fname}; using '.' as ID")

        # iterate rows
        for _, row in df.iterrows():
            chrom = norm_chrom(row[chrom_col])
            pos = str(row[pos_col]).strip()
            if chrom == "" or pos == "" or pos in (".", "NA", "nan"):
                continue
            if id_col:
                raw_id = str(row.get(id_col, "")).strip()
                vid = raw_id if raw_id not in ("", ".", "NA", "nan") else "."
            else:
                vid = "."
            key = f"{chrom}:{pos}:{vid}"
            key_to_samples[key].add(sample)
            if key not in key_to_example_row:
                key_to_example_row[key] = {"chrom": chrom, "pos": pos, "id": vid}

    # Build summary rows
    summary = []
    for key, sampleset in key_to_samples.items():
        chrom, pos, vid = key.split(":", 2)
        count = len(sampleset)
        samples_list = sorted(sampleset)
        try:
            loc_val = int(pos)
        except Exception:
            loc_val = pos
        summary.append({
            "Chromosome": chrom,
            "Location": loc_val,
            "ID": vid,
            "Count": count,
            "Samples": ";".join(samples_list)
        })

    if not summary:
        print("No variants found across input files.", file=sys.stderr)
        sys.exit(1)
```

```
    # DataFrame and sorting: Count desc, Chrom sort key, Location asc
    sdf = pd.DataFrame(summary)
    sdf["Chrom_sort_key"] = sdf["Chromosome"].apply(chrom_sort_key)
    sdf["Loc_sort"] = sdf["Location"].apply(lambda x: int(x) if isinstance(x, int) or (isinstance(x, str)
and x.isdigit()) else 10**12)
    sdf = sdf.sort_values(by=["Count", "Chrom_sort_key", "Loc_sort"], ascending=[False, True,
True]).reset_index(drop=True)
    out_df = sdf[["Chromosome", "Location", "ID", "Count", "Samples"]]

    out_df.to_csv(OUTFILE, sep="\t", index=False)
    print(f"Wrote {len(out_df)} variants to {OUTFILE}")

if __name__ == "__main__":
    main()#!/bin/bash
# a search strategy to identify prostate cancer (PCa) somatic SNVs from variant calls
# download the Somatic variant list from ClinVar (https://www.ncbi.nlm.nih.gov/clinvar) by the
keyword "Prostate Cancer"
# the ClinVar somatic list ----> clinvar_somatic_variants.txt

cut -f8,9,12,16 clinvar_somatic_variants.txt \
     > clinvar_somatic_variants_dbsnp.txt     # Extract only Chromosome (GRCh38Chromsome),
position (GRCh38Location), rsID (dbSNP ID) and clinvar classification

# the output might contain some range for short variants
# the next section demarkate the start and end positions for small variants

################################################################################
(FORMAT)
##################################################################################

awk 'BEGIN{FS=OFS="\t"}
NR==1 {
  print "Chr","start","End","ID"
  next
}
{
  # read fields (robust to missing $3)
  chr = $1
  loc = $2
  id  = ($3=="" ? "." : $3)

  # trim whitespace
  gsub(/^[ \t]+|[ \t]+$/, "", chr)
  gsub(/^[ \t]+|[ \t]+$/, "", loc)
  gsub(/^[ \t]+|[ \t]+$/, "", id)

  # extract numbers from loc; handles "12345", "12345-12346", "12345 - 12346", etc.
  if (loc == "" ) {
    start = ""; end = ""
  } else {
    n = split(loc, a, /[^0-9]+/)
    # the split above may create empty elements; find first two numeric tokens
```

```
    start=""; end=""
    for(i=1;i<=n;i++){
      if(a[i] ~ /^[0-9]+$/) {
        if(start=="") start=a[i]
        else if(end=="") end=a[i]
      }
    }
    if(start=="" ) start = loc      # fallback: keep original text
    if(end=="" ) end = start
  }

  print chr, start, end, id
}' clinvar_somatic_variants_dbsnp.txt > clinvar_somatic_chr_start_end_id.txt

# FINAL: search for matched somatic records from the formatted clinvar list to the target VCF file
# search key: chr:pos:id
# if the rsID is missing in the target VCF then automatically reverts back to chr:pos and record all possible REF and ALT alleles in VCF format
#################################################################################
#################################################################################
#######

awk -F'\t' 'BEGIN { OFS = FS = "\t" }
NR==FNR {
    # Skip header in TSV if present (assumes header label "Chr"; if not add "chr")
    if (FNR==1 && $1 ~ /^Chr$/) next
    chr = $1; sub(/^chr/, "", chr)      # normalize TSV chr (strip leading "chr")
    pos = $2
    rsid = ($4 == "" ? "." : $4)       # use "." for missing RSID
    clinmap[chr "_" pos] = rsid
    next
}
# Now processing VCF file
/^##/ { print; next }                  # print meta-lines unchanged
/^#CHROM/ { print; next }              # print header line once
{
    chr = $1
    tmp = chr; sub(/^chr/, "", tmp)    # normalize VCF chr for matching
    key = tmp "_" $2

    # Only print variants that are present in the TSV (match by chr:pos)
    if (key in clinmap) {
        if (clinmap[key] != ".") {
            $3 = clinmap[key]        # populate ID only if TSV has rsID
        }
        print
    }
    # else skip (do not print unmatched variant lines)
}' clinvar_somatic_chr_start_end_id.tsv WES_LPS.vt.vcf > WES_LPS_somatic_vt.vcf#!/bin/bash
# a bash script for identifying gnomAD annotated structural variants
```

```
zcat gnomad.v4.1.sv.sites.bed.gz | cut -f1,2,3,5 | awk 'BEGIN{OFS="\t"} $1!~/^#/ {print
$1,$2,$3,$4}' | sort -k1,1V -k2,2n > gnomad4.sv.bed
```

```
bcftools query -f '%CHROM\t%POS\t%ID\t%REF\t%ALT\n' WES.vt_indels.vcf.gz \
  | awk 'BEGIN{OFS="\t"}{print $1, $2-1, $2, $2, $3, $4, $5}' \
  | sort -k1,1V -k2,2n \
  | bedtools intersect -a - -b gnomad4.sv.bed -wa -wb \
  | awk 'BEGIN{OFS="\t"; print "CHROM","POS","ID","REF","ALT","END","SVTYPE"} {print
$1,$4,$5,$6,$7,$9,$10}' \
  > matched_vcf_with_gnomad_svinfo.tsv
mv matched_vcf_with_gnomad_svinfo.tsv WES.vt_indels.point.bed
```
